# Tartaric Acid as an Interfacial Bridge: Constructing a Tight SnS_2_/CdIn_2_S_4_ Junction for Efficient Photoelectrochemical Water Oxidation

**DOI:** 10.3390/ma19214762

**Published:** 2026-11-09

**Authors:** Jiangchuan Liu, Xiaoxu Wang, Yupeng He, Bolin Wang, Guoquan Yang, Zhihui Chen, Yinlin Shen

**Affiliations:** 1 School of Materials Science and Engineering, Jiangsu Province Engineering Research Center of Intelligent Manufacturing Technology for the New Energy Vehicle Power Battery, Changzhou University, Changzhou 213164, China; 2 Department of Chemistry, City University of Hong Kong, Hong Kong 999077, China

**Keywords:** photoelectrochemical, photoanode, CdIn_2_S_4_, SnS_2_/CdIn_2_S_4_ heterojunction, tartaric acid, charge separation

## Abstract

Strengthening the interfacial interaction of heterojunction photoanodes is an important route to efficient photoelectrochemical (PEC) water splitting. Herein, a SnS_2_/CdIn_2_S_4_ heterojunction photoanode is fabricated by a two-step solvothermal method, in which tartaric acid (TA) acts as an interfacial bridge to regulate the growth of SnS_2_ on trapezoid-like CdIn_2_S_4_. The optimized photoanode delivers a photocurrent density of 5.93 mA cm^−2^ at 1.23 V vs. RHE, approximately 6.9 times that of pristine CdIn_2_S_4_ (0.86 mA cm^−2^), with a maximum applied bias photon-to-current efficiency (ABPE) of 2.81%. Electrochemical impedance spectroscopy shows a reduced charge-transfer resistance, and time-resolved photoluminescence reveals a prolonged carrier lifetime. Density functional theory calculations indicate the proposed interfacial charge-transfer mechanism: the work-function difference between SnS_2_ (6.12 eV) and CdIn_2_S_4_ (3.95 eV) drives interfacial electron transfer from CdIn_2_S_4_ to SnS_2_, the charge density difference reveals electron accumulation on the SnS_2_ side and depletion on the CdIn_2_S_4_ side, with the interfacial charge transfer increasing from 0.33 to 0.39 e^−^ after TA bridging, and the projected density of states (PDOS) evidences a strong electronic interaction across the junction. This organic-molecule-assisted interfacial bridging offers a facile route for designing the interfaces of sulfide photoanodes.

## 1. Introduction

The growing world population and energy consumption have intensified the search for clean alternatives to fossil fuels [1,2,3,4,5]. Photocatalytic CO_2_ reduction and photoelectrochemical (PEC) water splitting, which convert and store intermittent solar energy as chemical fuels, are regarded as two of the most promising solutions [6,7,8]. Because water oxidation on the photoanode involves a four-hole process with sluggish kinetics, the overall efficiency of a PEC cell is generally limited by the photoanode, which must separate the photogenerated carriers efficiently and transfer the holes to the electrolyte [9,10,11]. Developing photoanodes with fast charge separation and transfer is therefore essential. Compared with metal oxides, metal sulfide semiconductors feature narrower bandgaps and longer carrier diffusion lengths because their valence bands are mainly composed of S 3p orbitals, which makes them attractive candidates for visible-light-driven PEC applications [12]. Cadmium indium sulfide (CdIn_2_S_4_), a ternary chalcogenide with a cubic spinel structure, has a suitable direct bandgap of about 2.2 eV, good visible-light absorption and high structural stability [13,14,15]. Nevertheless, pristine CdIn_2_S_4_ suffers from severe recombination of photogenerated carriers and sluggish surface water-oxidation kinetics, which restricts its PEC performance [13,14]. Tin disulfide (SnS_2_) is a two-dimensional layered semiconductor in which adjacent S-Sn-S slabs interact through van der Waals forces, giving rise to a large interlayer spacing. It combines a bandgap of 2.0–2.6 eV with strong optical anisotropy and good electronic conductivity; Sn and S are also earth-abundant elements [12]. These features make SnS_2_ a promising partner semiconductor for CdIn_2_S_4_.

Constructing heterojunctions is an effective strategy to accelerate the transfer and separation of photogenerated charges and to enhance the stability of photoanodes [9,16]. When two semiconductors with different work functions come into contact, electrons flow from the lower-work-function component to the higher-work-function one, and the resulting interfacial electric field drives photogenerated electrons and holes to opposite sides of the junction [17,18,19]. Chen et al. [20] showed by DFT calculations that a two-dimensional ZnS/SnS_2_ heterojunction is stable and has higher carrier mobility and stronger light absorption than the isolated monolayers, because the interfacial built-in field promotes the separation of photogenerated electron–hole pairs. However, the separation efficiency of a heterojunction is highly sensitive to the quality of the heterointerface; loose contact and uncontrolled overlayer growth lengthen the carrier transport path and introduce interfacial recombination centers [21,22]. Constructing a tight and well-coupled sulfide/sulfide interface therefore remains a key challenge for CdIn_2_S_4_-based photoanodes.

Small organic molecules bearing hydroxyl and carboxyl groups can coordinate metal ions and thereby control the nucleation and growth rate of nanocrystals, modulate the exposure of crystal facets and improve the contact between phases [23,24,25]. Tartaric acid (TA), an inexpensive and green dicarboxylic acid containing two hydroxyl and two carboxyl groups, has been shown to complex with metal centers on a photoanode surface and modulate its interfacial charge behavior [26]. Moreover, TA-assisted hydrothermal growth has been employed to prepare cubic ZnIn_2_S_4_ films [27]. In this work, we report a TA-regulated SnS_2_/CdIn_2_S_4_ heterojunction photoanode grown on fluorine-doped tin oxide (FTO) through a simple two-step solvothermal route. The optimized photoanode achieves a photocurrent density of 5.93 mA cm^−2^ at 1.23 V vs. RHE, approximately 6.9 times that of pristine CdIn_2_S_4_ (0.86 mA cm^−2^), and a maximum ABPE of 2.81%. TA bridges the SnS_2_ overlayer and the CdIn_2_S_4_ substrate and regulates the growth and facet exposure of SnS_2_. By increasing the carrier density, and accelerating the interfacial hole transfer, it promotes the spatial separation of photogenerated electron–hole pairs. The photocurrent density and ABPE achieved in this work compare favorably with those of recently reported CdIn_2_S_4_-based heterojunction photoanodes. This work provides a simple way to regulate carrier transport at heterojunction interfaces for efficient PEC water splitting.

## 2. Materials and Methods

### 2.1. Synthesis of the CdIn_2_S_4_ Photoanode

The FTO glass was ultrasonicated in acetone, deionized water and ethanol for 20 min each and dried under N_2_ flow. CdCl_2_ (0.017 M, Aladdin, Shanghai, China), InCl_3_ (0.034 M, Aladdin, Shanghai, China) and CH_4_N_2_S (0.27 M, Aladdin, Shanghai, China) were dissolved in 35 mL of aqueous solution. The clean FTO glass (Jinge, Wuhan, China) was placed in a Teflon-lined autoclave (Chenghua, Suzhou, China) with the conductive side facing the wall and held at 160 °C for 12 h. After the autoclave had cooled naturally to room temperature, the film was rinsed with deionized water and ethanol (Aladdin, Shanghai, China) and dried at 60 °C, and finally annealed at 400 °C for 1 h in an Ar/H_2_ (5%) atmosphere. The resulting electrode is denoted as the pristine CdIn_2_S_4_ photoanode.

### 2.2. Synthesis of the SnS_2_/CdIn_2_S_4_ Photoanode

SnS_2_ was grown on CdIn_2_S_4_ by a solvothermal method. SnCl_4_ (0.008 M, Aladdin, Shanghai, China) and CH_4_N_2_S (0.016 M) were dissolved in 35 mL of ethanol and stirred for 30 min. The solution and the CdIn_2_S_4_ photoanode were transferred into an autoclave and held at 75 °C for 2 h. The electrode was then taken out, rinsed with deionized water and ethanol, dried naturally, and finally annealed in air at 300 °C for 1 h to obtain the SnS_2_/CdIn_2_S_4_ photoanode.

### 2.3. Synthesis of the TA-SnS_2_/CdIn_2_S_4_ Photoanode

The TA-SnS_2_/CdIn_2_S_4_ photoanode was prepared by the same procedure as SnS_2_/CdIn_2_S_4_, except that TA (12 mM, Aladdin, Shanghai, China) was dissolved in the precursor solution before the solvothermal reaction.

### 2.4. Characterization

The surface and cross-sectional morphologies were characterized by scanning electron microscopy (SEM, JSM-6360LA, JEOL, Tokyo, Japan). The microstructure was examined by transmission electron microscopy (TEM, JEOL 2100, 200 kV, JEOL, Tokyo, Japan) equipped with energy-dispersive X-ray spectroscopy (EDS). The crystallinity was analyzed by X-ray diffraction (XRD, D/MAX 2500, Rigaku, Tokyo, Japan; Cu Kα radiation, λ = 0.15418 nm, 10–80°), and Raman spectra were recorded on a LabRAM HR Evolution spectrometer (HORIBA, Kyoto, Japan) with a 532 nm excitation laser. The chemical composition and elemental states were determined by X-ray photoelectron spectroscopy (XPS, ESCALab 250Xi, Thermo Fisher Scientific, Waltham, MA, USA), with all binding energies calibrated to the C 1s peak at 284.8 eV. UV-vis diffuse reflectance spectra (DRS) were collected on a UV-3600i Plus spectrophotometer (Shimadzu, Kyoto, Japan, 200–800 nm) with FTO as the reference. Steady-state photoluminescence (PL) spectra were recorded on an FL6500 fluorometer (PerkinElmer, Waltham, MA, USA, excitation at 400 nm), and time-resolved photoluminescence (TRPL) spectra were measured on an FLS1000 spectrometer (Edinburgh Instruments, Livingston, UK).

### 2.5. PEC Measurements

The PEC performance was evaluated in a conventional three-electrode system on an LK5600 photoelectrochemical workstation (Lanlike, Tianjin, China), in which the as-prepared photoanode, a saturated Ag/AgCl electrode and a Pt plate served as the working, reference and counter electrodes, respectively. An AM 1.5G solar simulator (100 mW cm^−2^, Enlitech, Guangdong, China) was used as the light source, and the electrolyte was an aqueous solution containing 0.35 M Na_2_SO_3_ and 0.25 M Na_2_S. The potential of the working electrode (with respect to Ag/AgCl) was converted to the reversible hydrogen electrode (RHE) by the Nernst equation:

(1) $$E_{\mathrm{RHE}}(V)\;=\;E_{\mathrm{Ag}/\mathrm{AgCl}\;}+\;0.059\;\times \;\mathrm{pH}\;+\;E_{\mathrm{Ag}/\mathrm{AgCl}}^{0}$$

where *E*_RHE_ is the voltage value converted to RHE, *E*_Ag/AgCl_ is the voltage value obtained from laboratory electrode testing, and *E*^0^_Ag/AgCl_ is the standard potential of a saturated Ag/AgCl electrode at room temperature (0.1976 V). The applied bias photon-to-current efficiency (ABPE) was calculated using the following equation:

(2) $$\mathrm{ABPE}(\%)=\frac{J_{P}\times (1.23-E_{\mathrm{RHE}})}{P_{\mathrm{light}}}\times 100\%$$

where *J*_P_ is the photocurrent density, *E*_RHE_ is the applied bias voltage, and P_light_ is the incident optical power density.

Linear sweep voltammetry (LSV) was performed at a scan rate of 30 mV s^−1^ under illumination. Electrochemical impedance spectroscopy (EIS) was carried out at the open-circuit potential under illumination with an amplitude of 10 mV over the frequency range of 0.1–100 kHz and fitted to the equivalent circuit of Rs(QRct). Mott–Schottky (M-S) plots were recorded in the dark at 1000 Hz with an amplitude of 10 mV. Transient photocurrent (I-t) curves were collected at 1.23 V vs. RHE under chopped illumination for 300 s with an interval of 15 s, and open-circuit potential (OCP) curves were measured for 300 s with the light and dark conditions alternated every 100 s, starting in the dark. The IPCE was measured at selected wavelengths and at 1.23 V vs. RHE under monochromatic illumination. Monochromatic light at wavelength λ (nm) was generated using long-wavelength filters, and the output power was measured using a photodiode detector.

### 2.6. Computational Details

All DFT calculations in this work were performed with the CP2K package (version 2023), in which the electronic structure is described by a mixed Gaussian and plane-wave (GPW) scheme. The valence electrons were represented with DZVP-MOLOPT-SR-GTH basis sets, while the core electrons were replaced by Goedecker–Teter–Hutter (GTH) pseudopotentials. An energy cutoff of 500 Ry was applied to the auxiliary plane-wave basis. Geometry optimization was conducted with the BFGS algorithm until the residual force on every atom dropped below 4.5 × 10^−4^ hartree bohr^−1^. A vacuum layer of about 20 Å was inserted along the surface normal to remove the spurious interaction between periodically repeated slabs, and the in-plane lattice mismatch of the constructed heterostructure models was kept below 5% in both lateral directions.

## 3. Results and Discussion

**Figure 1:**
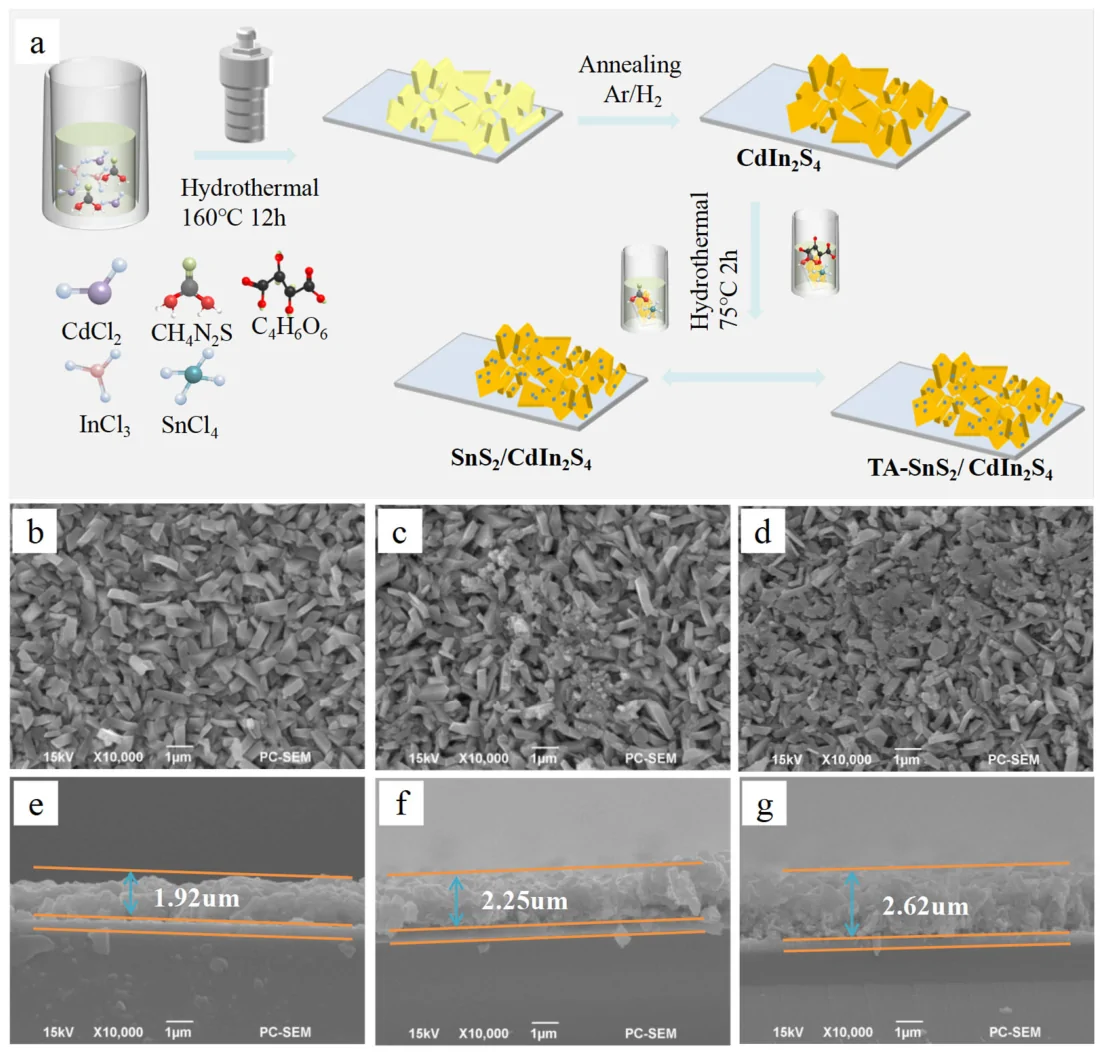
(**a**) Schematic illustration of the fabrication process of the TA-SnS_2_/CdIn_2_S_4_ photoanode; (**b**–**d**) surface and (**e**–**g**) cross-sectional SEM images of the (**b**,**e**) CdIn_2_S_4_, (**c**,**f**) SnS_2_/CdIn_2_S_4_ and (**d**,**g**) TA-SnS_2_/CdIn_2_S_4_ photoanodes.

Figure 1a illustrates the fabrication of the TA-SnS_2_/CdIn_2_S_4_ photoanode. CdIn_2_S_4_ is first grown on FTO by hydrothermal deposition at 160 °C for 12 h and annealed in Ar/H_2_, and SnS_2_ is then deposited on the CdIn_2_S_4_ surface by a second solvothermal step at 75 °C for 2 h in the presence of TA. Subsequently, the morphologies of the three photoanodes were examined by SEM (Figure 1b–g). Pristine CdIn_2_S_4_ consists of trapezoid-like plates that form a compact film with a thickness of 1.92 μm (Figure 1b,e). After the second solvothermal step, fragment- and granule-like deposits appear on the CdIn_2_S_4_ surface (Figure 1c), and the cross-section thickens to 2.25 μm (Figure 1f), an increase of about 0.33 μm, suggesting the formation of SnS_2_ on CdIn_2_S_4_. In addition, with TA added, the deposits grow into larger plate-like structures while the underlying CdIn_2_S_4_ keeps its morphology and size (Figure 1d), and the cross-section further thickens to 2.62 μm (Figure 1g), an increase of 0.37 μm. TA thus regulates the growth morphology of SnS_2_ and yields a thicker overlayer, which offers a larger interfacial contact area and anchors SnS_2_ more tightly on CdIn_2_S_4_, with TA acting as an interfacial bridge.

**Figure 2:**
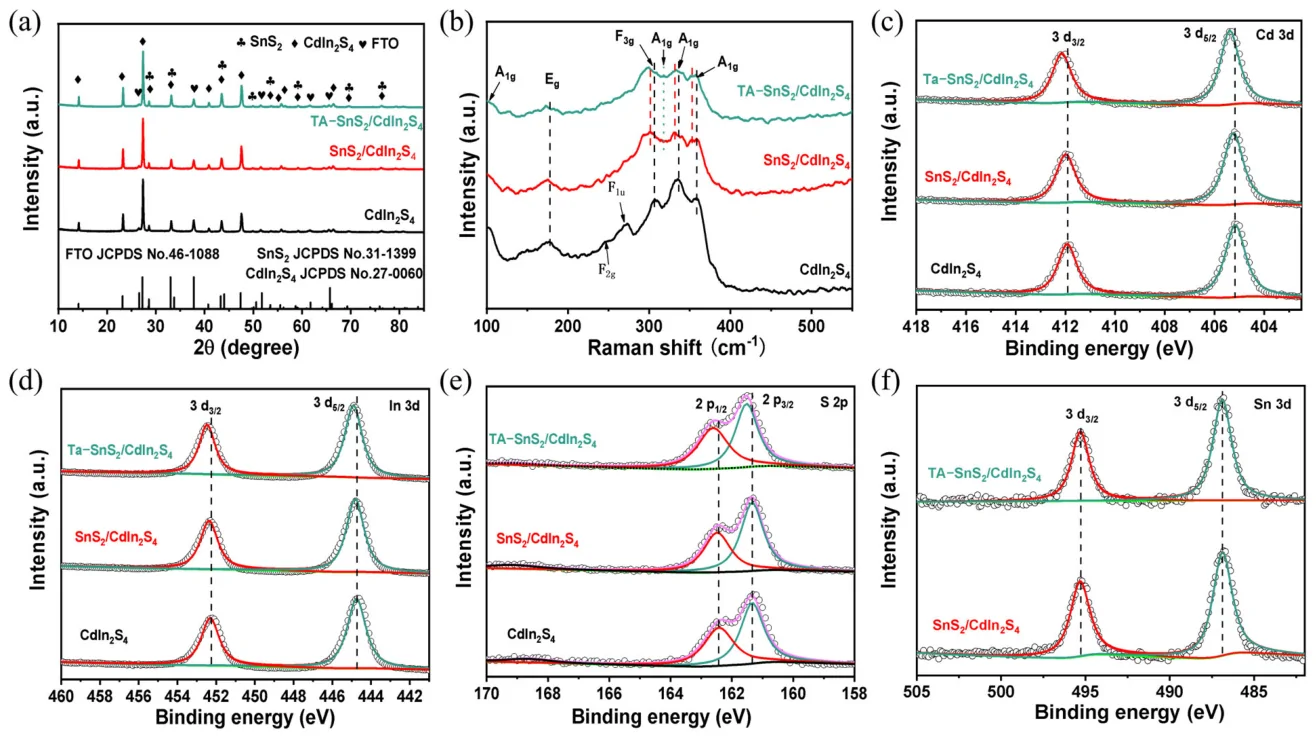
(**a**) XRD patterns, (**b**) Raman spectra and (**c**–**f**) high-resolution XPS spectra of (**c**) Cd 3d, (**d**) In 3d, (**e**) S 2p and (**f**) Sn 3d of the CdIn_2_S_4_, SnS_2_/CdIn_2_S_4_ and TA-SnS_2_/CdIn_2_S_4_ photoanodes.

The XRD patterns in Figure 2a confirm the phases of the photoanodes. Besides the signals from the FTO substrate, the diffraction peaks can be indexed to the cubic phase of CdIn_2_S_4_ (JCPDS No. 27-0060), and additional reflections of the hexagonal phase of SnS_2_ (JCPDS No. 31-1399) emerge for the composite photoanodes, confirming the formation of the SnS_2_/CdIn_2_S_4_ composite. The reflections of the CdIn_2_S_4_ (311), (400) and (511) planes at 27.249°, 33.001° and 43.318° are weakened after SnS_2_ growth, probably because the overlayer partially screens the underlying CdIn_2_S_4_ surface. After TA is added, these CdIn_2_S_4_ reflections recover in intensity, while the (101) and (102) reflections of SnS_2_ at 33.019° and 44.019° are enhanced. TA therefore regulates the growth planes of SnS_2_ and re-exposes the active facets of CdIn_2_S_4_. In addition, the Raman spectra (Figure 2b) confirm the cubic spinel structure, in good agreement with the XRD results. CdIn_2_S_4_ exhibits peaks at 102, 177, 247, 273, 306, 335 and 359 cm^−1^, assigned to the A_1g_, E_g_, F_2g_, F_1u_, F_3g_, A_1g_ and A_1g_ modes, respectively [13,15]. For SnS_2_/CdIn_2_S_4_ the peaks broaden and weaken, and a weak A1g mode of hexagonal SnS_2_ appears at 316 cm^−1^, probably because the small amount of SnS_2_ is partly masked by the CdIn_2_S_4_ matrix. After coupling with SnS_2_, the F_3g_ and A_1g_ modes of CdIn_2_S_4_ shift to lower frequencies, suggesting that the interfacial groups are perturbed when the heterojunction forms; that is, SnS_2_ couples to the CdIn_2_S_4_ surface chemically rather than physically.

To further examine the chemical states and the interfacial electronic interaction, XPS characterization was employed (Figure 2c–f). The XPS survey spectra (Figure S1) identify Cd, In, S, Sn and O, along with the adventitious C 1s signal used for calibration, and no other elements were detected, consistent with the results of elemental mapping. For CdIn_2_S_4_ and SnS_2_/CdIn_2_S_4_, the Cd 3d doublet appears at 411.9 and 405.2 eV, assigned to Cd 3d_3/2_ and Cd 3d_5/2_ of Cd^2+^, and shifts to 412.1 and 405.4 eV for TA-SnS_2_/CdIn_2_S_4_ (Figure 2c). The In 3d doublet, located at 452.3 and 444.7 eV, corresponding to In 3d_3/2_ and In 3d_5/2_ of In^3+^, moves to 452.5 and 444.9 eV after TA is introduced (Figure 2d). The S 2p peaks at 162.4 and 161.3 eV (S^2−^, 2p_1/2_ and 2p_3/2_) shift to 162.6 and 161.5 eV (Figure 2e). In contrast, the Sn 3d doublet at 495.3 and 486.9 eV (Sn 3d_3/2_ and Sn 3d_5/2_ of Sn^4+^) is nearly identical for SnS_2_/CdIn_2_S_4_ and TA-SnS_2_/CdIn_2_S_4_ (Figure 2f). The binding energies of Cd 3d, In 3d and S 2p barely change from CdIn_2_S_4_ to SnS_2_/CdIn_2_S_4_, indicating that the growth of SnS_2_ alone does not alter the chemical environment of CdIn_2_S_4_. The positive shifts observed only after TA addition suggest a decreased electron density around CdIn_2_S_4_, which indicates that the carboxyl groups of TA promote the interfacial charge redistribution. Consequently, the directional transfer of holes is proposed to promote the spatial separation of photogenerated electron–hole pairs [23,28].

**Figure 3:**
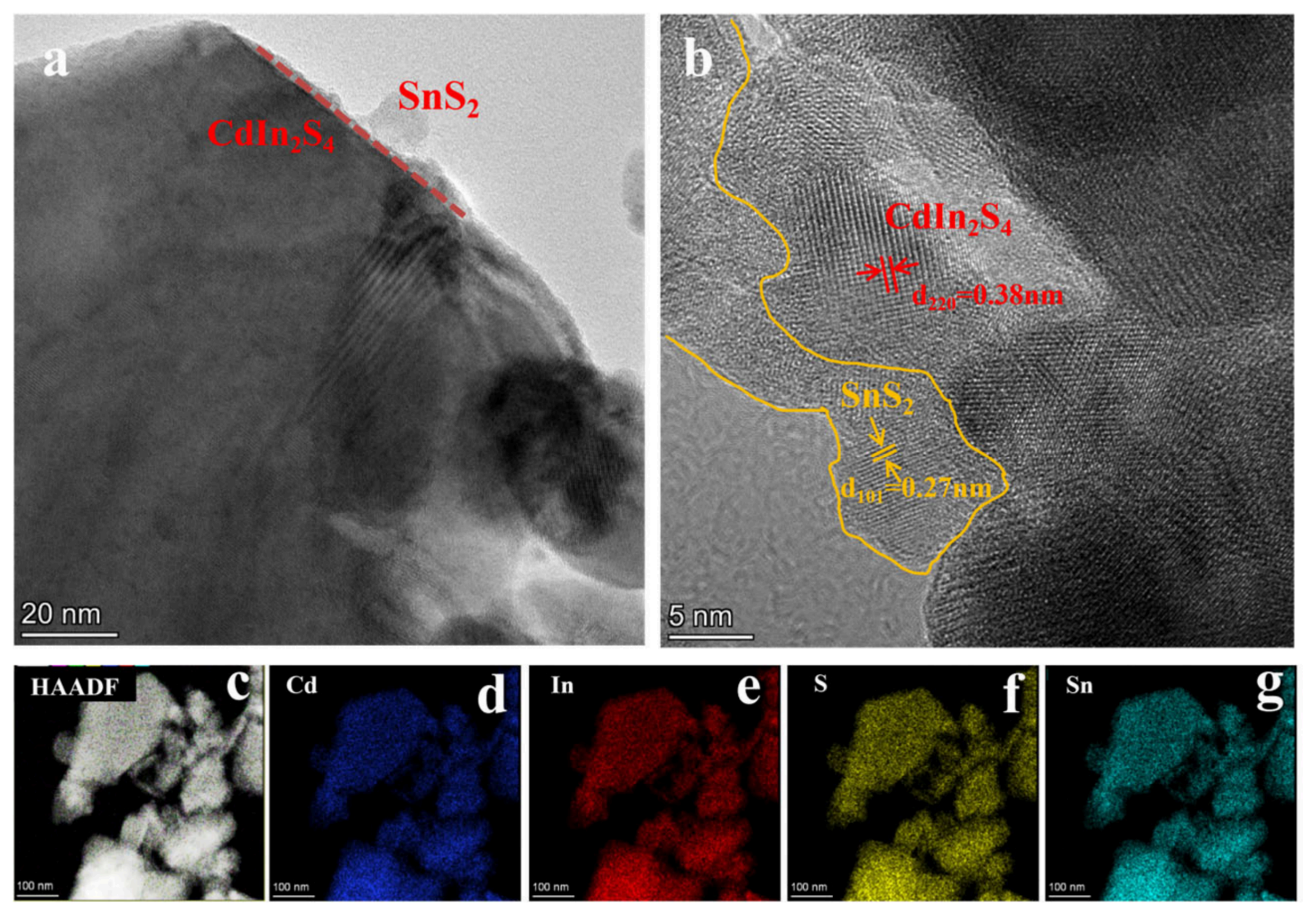
(**a**) TEM image, (**b**) HRTEM image, (**c**) HAADF-STEM image and (**d**–**g**) Cd, In, S and Sn elemental mapping images of the TA-SnS_2_/CdIn_2_S_4_ photoanode.

The microstructure of TA-SnS_2_/CdIn_2_S_4_ was further examined by TEM (Figure 3). A clear boundary between the two phases is observed in Figure 3a, indicating that SnS_2_ grows on the CdIn_2_S_4_ surface. The high-resolution image in Figure 3b shows lattice fringes of 0.38 nm and 0.27 nm, which correspond to the (220) plane of cubic CdIn_2_S_4_ and the (101) plane of hexagonal SnS_2_, respectively, confirming the intimate contact between the two semiconductors. The HAADF-STEM image and the EDS elemental mappings (Figure 3c–g) show that Cd, In, S and Sn are uniformly distributed over the whole region, further verifying the successful growth of SnS_2_ on the photoanode.

**Figure 4:**
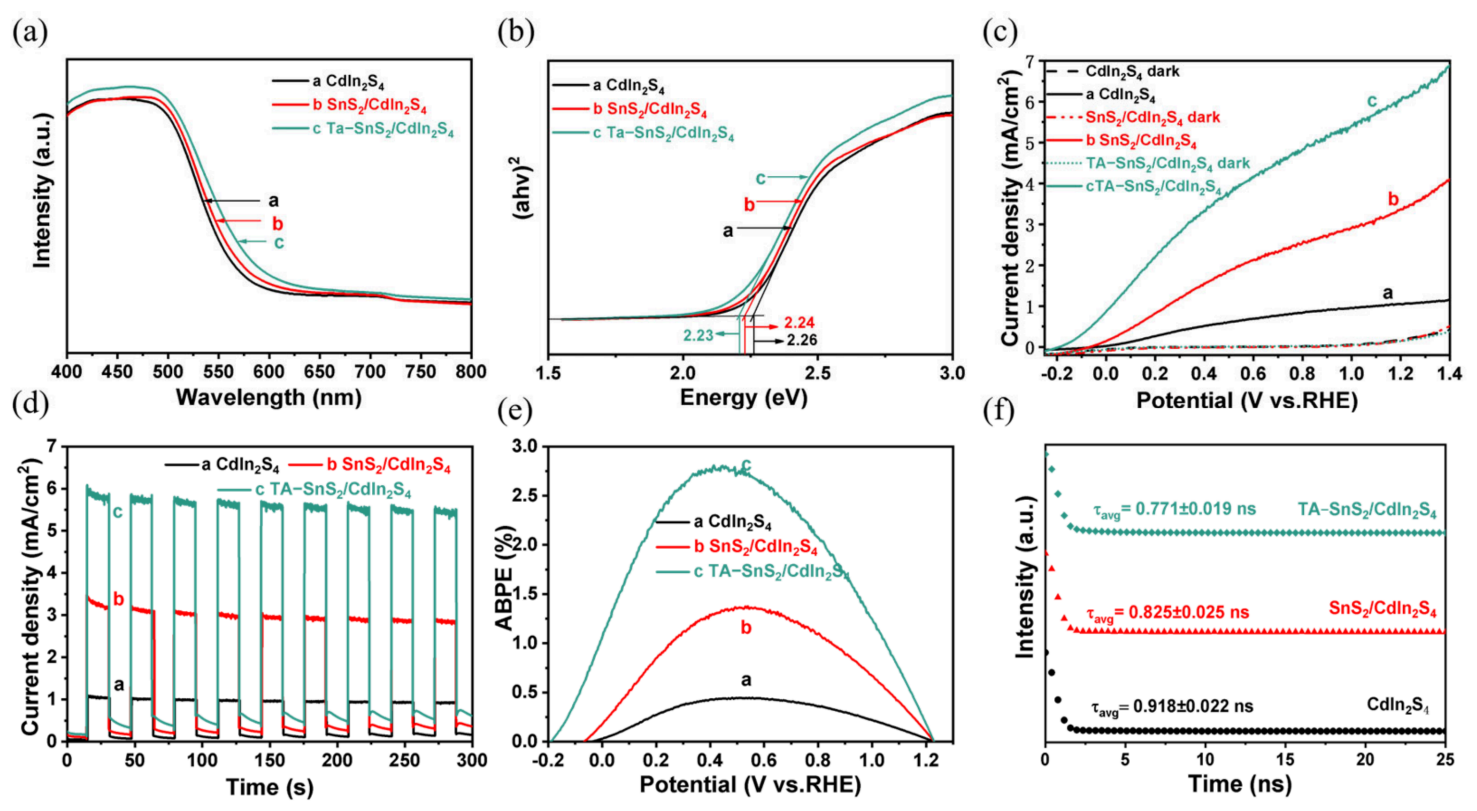
(**a**) UV-vis absorption spectra (FTO as reference), (**b**) corresponding Tauc plots, (**c**) LSV curves, (**d**) transient photocurrent responses at 1.23 V vs. RHE, (**e**) ABPE curves and (**f**) TRPL decay curves of the CdIn_2_S_4_, SnS_2_/CdIn_2_S_4_ and TA-SnS_2_/CdIn_2_S_4_ photoanodes.

Subsequently, the light-harvesting ability was characterized by UV-vis spectroscopy with FTO as the reference (Figure 4a). Pristine CdIn_2_S_4_ shows a strong absorption edge at about 580 nm. After SnS_2_ modification, the edge slightly red-shifts to about 600 nm, indicating that the heterojunction broadens the visible-light response, although the absorbance changes little because of the small SnS_2_ loading. With TA, the absorbance in the visible region increases and the edge further red-shifts to about 640 nm, which is ascribed to the larger SnS_2_ nanoplates that enhance the light harvesting within the film. The bandgaps derived from the Tauc plots (Figure 4b) are 2.26, 2.24 and 2.23 eV for CdIn_2_S_4_, SnS_2_/CdIn_2_S_4_ and TA-SnS_2_/CdIn_2_S_4_, respectively. The narrowing is slight because the two sulfides have similar bandgaps of 2.0–2.4 eV, but it still reflects the formation of the heterojunction. The red-shift in the absorption onset with TA mainly originates from the enhanced light scattering of the larger SnS_2_ nanoplates, whereas the Tauc analysis reflects the direct band-to-band transition and is only weakly affected by the scattering tail.

The PEC water-splitting activities were then measured in an aqueous solution of 0.35 M Na_2_SO_3_ and 0.25 M Na_2_S under simulated solar illumination. The SnS_2_ deposition time, the annealing temperature and the TA concentration were first optimized by LSV (Figures S2–S4), and the optimal conditions of 2 h (Figure S2), 300 °C (Figure S3) and 12 mM (Figure S4) were adopted. As shown in Figure 4c, the photocurrent densities at 1.23 V vs. RHE (dark currents subtracted) are 0.86, 3.24 and 5.93 mA cm^−2^ for CdIn_2_S_4_, SnS_2_/CdIn_2_S_4_ and TA-SnS_2_/CdIn_2_S_4_, respectively, corresponding to enhancements of about 3.8 and 6.9 times after the SnS_2_ coupling and the TA regulation. We attribute the TA-induced enhancement to the exposure of more active facets, the strengthened visible-light absorption and the tighter interface, which shortens the carrier transport distance and lowers the interfacial charge-transfer barrier at the electrode/electrolyte boundary. Meanwhile, the chopped I-t curves recorded at 1.23 V vs. RHE (Figure 4d) show prompt and reproducible responses to the on/off switching of light, and the photocurrent densities of all three photoanodes remain stable over 300 s, consistent with the LSV results.

As shown in Figure 4e, the maximum ABPE values are 0.45%, 1.38% and 2.81% for CdIn_2_S_4_, SnS_2_/CdIn_2_S_4_ and TA-SnS_2_/CdIn_2_S_4_ at 0.56, 0.54 and 0.41 V vs. RHE, respectively. The ABPE values of SnS_2_/CdIn_2_S_4_ and TA-SnS_2_/CdIn_2_S_4_ are 3.1 and 6.3 times that of pristine CdIn_2_S_4_, respectively. The values for the TA-SnS_2_/CdIn_2_S_4_ electrode also surpass most results reported for CdIn_2_S_4_-based heterojunction photoanodes in the literature (Figure S5, Table S1) [29,30,31,32,33,34]. The gain mainly stems from suppressed charge recombination, faster charge transport and stronger visible-light absorption, all enabled by the heterojunction and TA regulation. The incident photon-to-current conversion efficiency (IPCE) further supports this conclusion (Figure S6). The IPCE in the 350–450 nm region increases from about 13% for CdIn_2_S_4_ to about 44% for SnS_2_/CdIn_2_S_4_ and about 72% for TA-SnS_2_/CdIn_2_S_4_. Because IPCE is normalized to the incident photon flux, this enhancement directly reflects the improved intrinsic photon-to-current conversion efficiency, indicating that the photocurrent gain mainly originates from the promoted interfacial charge separation and transfer. Integrating the IPCE spectra with the AM 1.5G photon flux reproduces the measured photocurrent densities within about 10%. The dynamics of the photogenerated carriers were probed by TRPL (Figure 4f), and decay curves were fitted with a biexponential model. The intensity-weighted average lifetime (Table S2) lengthens from 0.771 ± 0.019 ns for CdIn_2_S_4_ to 0.825 ± 0.022 ns for SnS_2_/CdIn_2_S_4_ and further to 0.918 ± 0.025 ns for TA-SnS_2_/CdIn_2_S_4_. The prolongation after heterojunction formation indicates that the junction inhibits the recombination of photogenerated electron–hole pairs [35], and the further prolongation with TA suggests that the TA-assisted growth additionally speeds up the carrier transport while suppressing the recombination.

**Figure 5:**
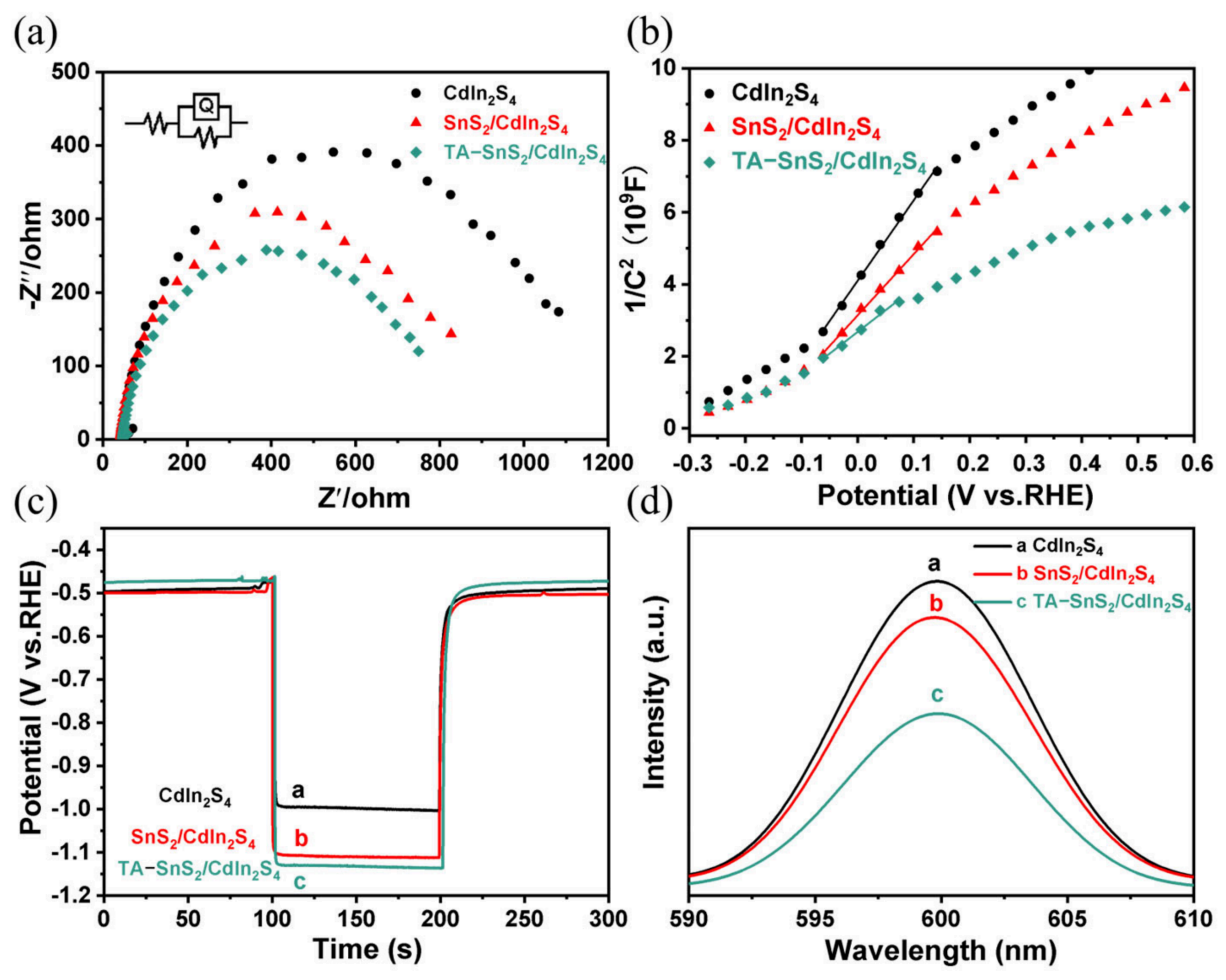
(**a**) EIS Nyquist plots under illumination (inset: equivalent circuit), (**b**) Mott–Schottky plots, (**c**) open-circuit potential curves and (**d**) steady-state PL spectra of the CdIn_2_S_4_, SnS_2_/CdIn_2_S_4_ and TA-SnS_2_/CdIn_2_S_4_ photoanodes.

Furthermore, EIS was measured under illumination and fitted to the equivalent circuit Rs(QRct) (Figure 5a). The charge-transfer resistance decreases from 1004 Ω for CdIn_2_S_4_ to 753.4 Ω for SnS_2_/CdIn_2_S_4_ and further to 670.8 Ω for TA-SnS_2_/CdIn_2_S_4_, indicating accelerated charge separation and transport after the heterojunction forms. The smallest semicircle of TA-SnS_2_/CdIn_2_S_4_ is probably related to the larger contact area and the tighter coupling between the two semiconductors achieved with the assistance of TA. Moreover, the Mott–Schottky plots (Figure 5b) show positive slopes for all samples, confirming the n-type character of the CdIn_2_S_4_-based photoanodes. TA-SnS_2_/CdIn_2_S_4_ has the smallest slope, implying the highest carrier density. The carrier densities of CdIn_2_S_4_, SnS_2_/CdIn_2_S_4_ and TA-SnS_2_/CdIn_2_S_4_ are calculated to be 7.33 × 10^19^, 9.63 × 10^19^ and 1.35 × 10^20^ cm^−3^, respectively. The heterojunction formation thus increases the carrier density, and TA further promotes it; the larger N_d_ also strengthens the electric field in the depletion region at the semiconductor/electrolyte interface, which favors the charge separation.

The interfacial charge separation was further evaluated by OCP measurements (Figure 5c). Upon illumination, the potentials of all three photoanodes shift negatively, and the light-minus-dark potential differences are 497, 601 and 645 mV for CdIn_2_S_4_, SnS_2_/CdIn_2_S_4_ and TA-SnS_2_/CdIn_2_S_4_, respectively; a larger difference means more efficient charge separation at the interface. The 104 mV increase from CdIn_2_S_4_ to SnS_2_/CdIn_2_S_4_ reflects the contribution of the heterojunction, and the further increase with TA reflects its role in promoting the interfacial charge transfer. Meanwhile, the steady-state PL emission of pristine CdIn_2_S_4_ (Figure 5d) is quenched after SnS_2_ loading and quenched further for TA-SnS_2_/CdIn_2_S_4_, confirming the progressively suppressed radiative recombination of the photogenerated carriers.

**Figure 6:**
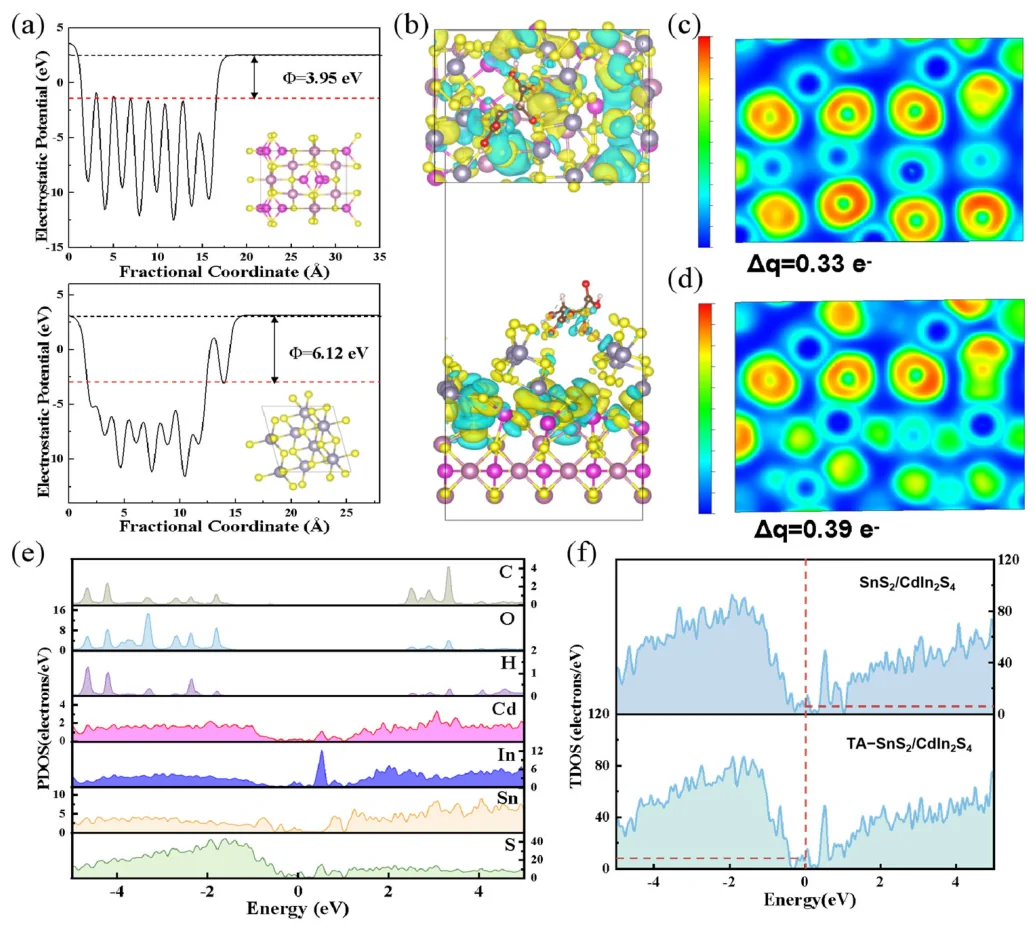
(**a**) Electrostatic potentials and work functions of CdIn_2_S_4_ and SnS_2_. (**b**) Differential charge density of TA-SnS_2_/CdIn_2_S_4_, in which the yellow and cyan isosurfaces represent charge accumulation and depletion, respectively. Electron localization function (ELF) of (**c**) SnS_2_/CdIn_2_S_4_ and (**d**) TA-SnS_2_/CdIn_2_S_4_; the interfacial charge transfer Δq increases from 0.33 to 0.39 e^−^ upon TA modification. (**e**) Projected density of states (PDOS) of TA-SnS_2_/CdIn_2_S_4_. (**f**) Total density of states (TDOS) of SnS_2_/CdIn_2_S_4_ and TA-SnS_2_/CdIn_2_S_4_, with the red dashed line denoting the Fermi level.

To investigate the effect of the TA-bridged interface on the electronic structure, DFT calculations were performed on the SnS_2_/CdIn_2_S_4_ and TA-SnS_2_/CdIn_2_S_4_ junction (Figure 6). The electrostatic potentials give work functions of 3.95 eV for CdIn_2_S_4_ and 6.12 eV for SnS_2_ (Figure 6a). Because electrons flow from the lower-work-function side to the higher-work-function side upon contact, the large work-function difference indicates electron transfer from CdIn_2_S_4_ to SnS_2_, leaving the SnS_2_ side electron-enriched and the CdIn_2_S_4_ side electron-depleted; the resulting interfacial electric field provides a plausible driving force for the proposed directional charge transfer across the heterointerface [17,18]. The interfacial charge redistribution of SnS_2_/CdIn_2_S_4_ (Figure S7) and TA-SnS_2_/CdIn_2_S_4_ (Figure 6b) is clearly characterized by the charge density difference, in which the yellow and cyan isosurfaces represent charge accumulation and depletion, respectively, indicating the directional electron transfer from CdIn_2_S_4_ to SnS_2_ across the heterointerface. The electrons thus accumulate on the SnS_2_ side of the junction and are depleted on the CdIn_2_S_4_ side, especially near the interface, rendering the SnS_2_ side electron-rich. This directional charge redistribution allows the photogenerated holes to be efficiently transferred across the junction and consumed by the S^2−^/SO_3_^2−^ electrolyte, while the electrons migrated toward the back contact and were collected by FTO, thus promoting the spatial separation of photogenerated electron–hole pairs; this is consistent with the positive XPS shifts in the CdIn_2_S_4_ elements [36] and the OCP results. To further probe the interfacial electronic interaction, the electron localization function (ELF) of SnS_2_/CdIn_2_S_4_ and TA-SnS_2_/CdIn_2_S_4_ was comparatively analyzed (Figure 6c,d), where the red and blue regions correspond to high and low degrees of electron localization, respectively. As quantified by Bader charge analysis, the interfacial charge transfer Δq increases from 0.33 e^−^ for SnS_2_/CdIn_2_S_4_ to 0.39 e^−^ for TA-SnS_2_/CdIn_2_S_4_, indicating that TA modification intensifies the interfacial charge redistribution. Compared with pristine SnS_2_/CdIn_2_S_4_, TA-SnS_2_/CdIn_2_S_4_ exhibits significantly enhanced electron delocalization within the CdIn_2_S_4_ component, which facilitates the rapid migration of photogenerated carriers across the heterointerface. Furthermore, the PDOS projected onto the constituent atoms of the TA-SnS_2_/CdIn_2_S_4_ junction is displayed in Figure 6e. The C, H, O, Sn, S, Cd and In states are located at coincident energies and exhibit similar peak shapes, indicating that the electronic states of TA and the two metal-sulfide components are hybridized with each other, which evidences the strong electronic interaction among TA, SnS_2_ and CdIn_2_S_4_ across the interface; such hybridized states also provide channels for the interfacial charge transfer. A similar hybridization among the Sn, S, Cd and In states is also observed in the PDOS of the bare SnS_2_/CdIn_2_S_4_ junction (Figure S8), confirming the intrinsic strong electronic coupling between SnS_2_ and CdIn_2_S_4_. As revealed by the TDOS in Figure 6f, the density of states at the Fermi level of TA-SnS_2_/CdIn_2_S_4_ (12.8 states eV^−1^) is clearly higher than that of the bare SnS_2_/CdIn_2_S_4_ (7.3 states eV^−1^), indicating that TA adsorption increases the carrier density near the Fermi level and thereby enhances the electrical conductivity of the heterojunction.

## 4. Conclusions

In summary, a TA-SnS_2_/CdIn_2_S_4_ heterojunction photoanode has been successfully fabricated through a two-step solvothermal method, in which tartaric acid (TA) plays a dual role: it regulates the growth and facet exposure of SnS_2_ on the trapezoid-like CdIn_2_S_4_, and it bridges the two sulfides into a tight and well-coupled junction. The experimental results can be rationalized by a proposed mechanism: the internal electric field built by the work-function difference is proposed to drive the photogenerated electrons toward the SnS_2_ side, while the holes are consumed at the CdIn_2_S_4_/electrolyte interface. Bader charge analysis further quantifies that TA bridging increases the interfacial charge transfer from 0.33 to 0.39 e^−^, thereby intensifying this electric field, and the higher Fermi-level TDOS of TA-SnS_2_/CdIn_2_S_4_ evidences its enhanced electrical conductivity; meanwhile, the coincident Sn, S, Cd and In PDOS confirms the strong interfacial electronic interaction. As a result, the enhanced interfacial charge-transfer kinetics and the prolonged carrier lifetime jointly promote the spatial separation of photogenerated electron–hole pairs. Benefiting from the bridged interface, the optimized photoanode delivers a photocurrent density of 5.93 mA cm^−2^ at 1.23 V vs. RHE (6.9 times that of pristine CdIn_2_S_4_), with a maximum ABPE of 2.81%. The TA-SnS_2_/CdIn_2_S_4_ photoanode reaches an IPCE of about 72% in the 350–450 nm region. This organic-molecule-assisted interfacial bridging strategy is complementary to cocatalyst-based interfacial regulation and provides a facile and general route for engineering the junction interfaces of sulfide photoanodes.

## Data Availability

The original contributions presented in this study are included in the article/Supplementary Materials. Further inquiries can be directed to the corresponding authors.

## Supplementary Materials

The following supporting information can be downloaded at: https://www.mdpi.com/article/10.3390/ma19214762/s1, Figure S1: XPS survey spectra of CdIn_2_S_4_, SnS_2_/CdIn_2_S_4_, and TA-SnS_2_/CdIn_2_S_4_ photoanodes; Figure S2: LSV curves of CdIn_2_S_4_ and SnS_2_/CdIn_2_S_4_ photoanodes prepared with different SnS_2_ deposition times; Figure S3: LSV curves of SnS_2_/CdIn_2_S_4_ photoanodes annealed at different temperatures; Figure S4: LSV curves of SnS_2_/CdIn_2_S_4_ photoanodes treated with different concentrations of TA (0, 6, 12, 24, and 48 mM); Figure S5: Comparison of the applied bias photon-to-current efficiency (ABPE) of the photoanode in this work with other reported CdIn_2_S_4_-based photoanodes; Figure S6: IPCE plots of CdIn_2_S_4_, SnS_2_/CdIn_2_S_4_ and TA-SnS_2_/CdIn_2_S_4_ photoanodes recorded at 1.23 V vs. RHE; Figure S7: DFT calculations of the SnS_2_/CdIn_2_S_4_ junction: (a) three-dimensional charge density difference (yellow, electron accumulation; cyan, electron depletion); (b) plane-averaged charge density difference along the z direction; Figure S8: Projected density of states (PDOS) of the SnS_2_/CdIn_2_S_4_ heterojunction; Table S1: Comparison of the photocurrent densities (at 1.23 V vs. RHE) and ABPE values of the TA-SnS_2_/CdIn_2_S_4_ photoanode with reported CdIn_2_S_4_-based photoanodes [29,30,31,32,33,34]; Table S2: Illustrative biexponential fitting parameters for TRPL decay profiles.
