# Supplementary material for: Tartaric Acid as an Interfacial Bridge: Constructing a Tight SnS2/CdIn2S4 Junction for Efficient Photoelectrochemical Water Oxidation
Source: Materials (Basel). 2026 Nov 9;19(21):4762. doi: 10.3390/ma19214762 (PMC13765179; doi:10.3390/ma19214762)
Supplement: Supplementary file 1 [file materials-19-04762-s001.zip › materials-4668541-supplementary.pdf]

Supplementary Materials

For

Tartaric Acid as an Interfacial Bridge: Constructing a Tight $SnS_2/CdIn_2S_4$ Junction for Efficient Photoelectrochemical Water Oxidation

Jiangchuan Liu[1], Xiaoxu Wang[1], Yupeng He[1], Bolin Wang[1], Guoquan Yang[1], Zhihui Chen[1,*], Yinlin Shen[2,*]

[1]*School of Materials Science and Engineering, Jiangsu Province Engineering Research Center of Intelligent Manufacturing Technology for the New Energy Vehicle Power Battery, Changzhou University, Changzhou 213164, China;*

[2]*Department of Chemistry, City University of Hong Kong, Hong Kong 999077, China;*

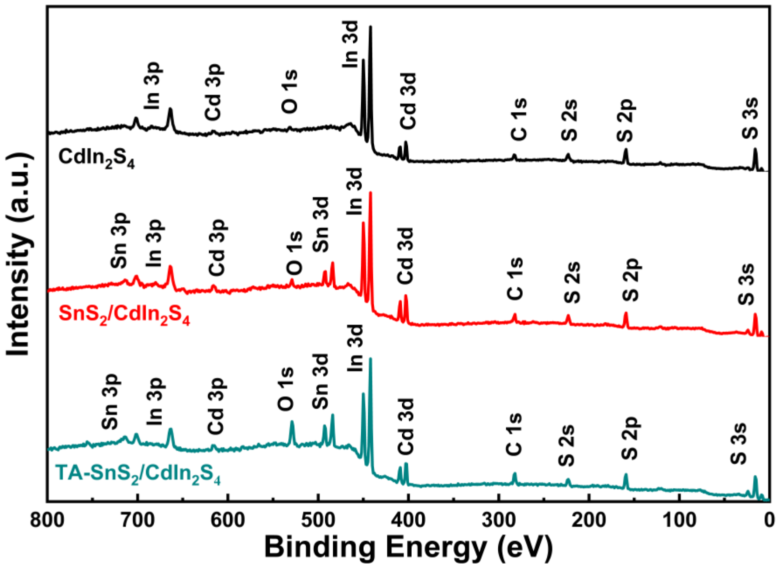

Figure S1. XPS survey spectra of $CdIn_2S_4$, $SnS_2/CdIn_2S_4$, and TA-$SnS_2/CdIn_2S_4$ photoanodes.

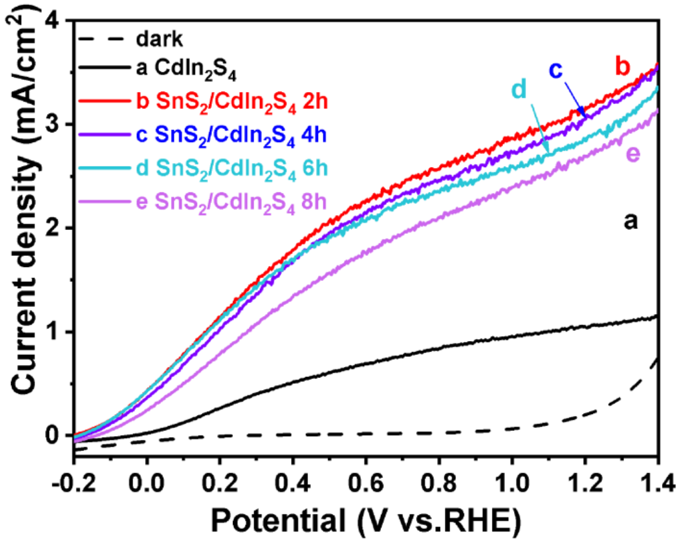

Figure S2. LSV curves of $CdIn_2S_4$ and $SnS_2/CdIn_2S_4$ photoanodes prepared with different SnS_2 deposition times.

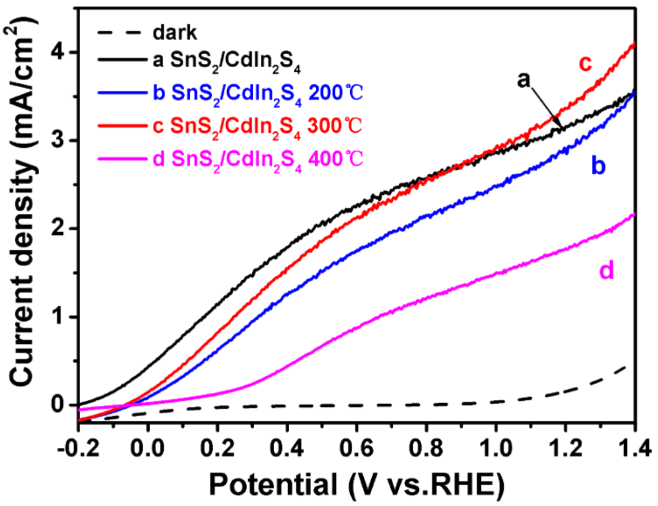

Figure S3. LSV curves of $SnS_2/CdIn_2S_4$ photoanodes annealed at different temperatures.

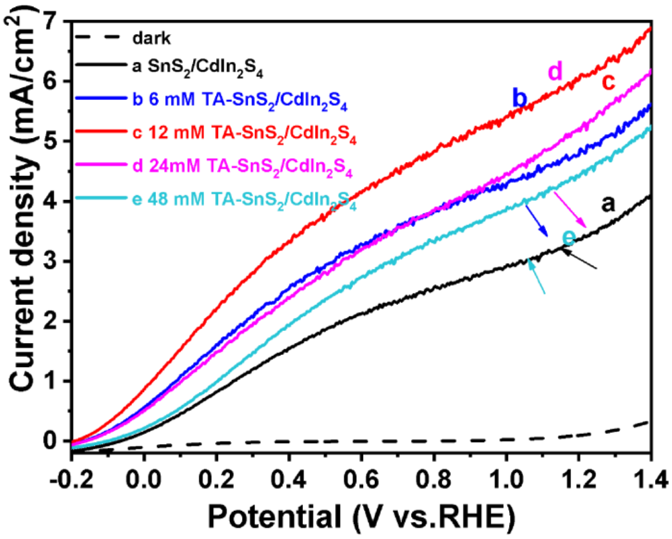

Figure S4. LSV curves of $SnS_2/CdIn_2S_4$ photoanodes treated with different concentrations of TA (0, 6, 12, 24, and 48 mM).

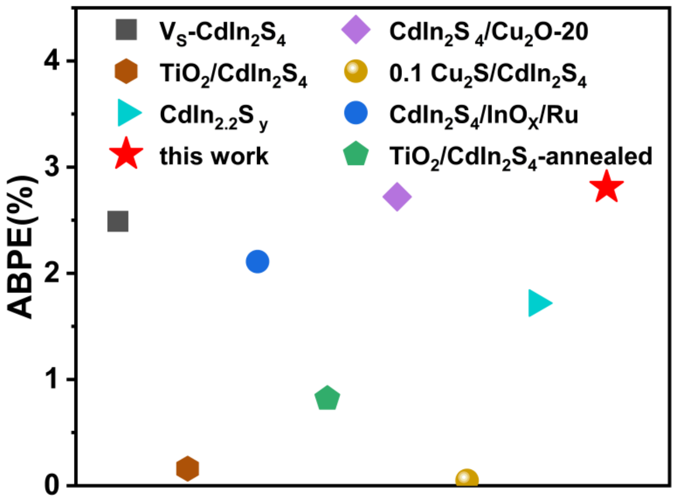

Figure S5. Comparison of the applied bias photon-to-current efficiency (ABPE) of the photoanode in this work with other reported $CdIn_2S_4$-based photoanodes.

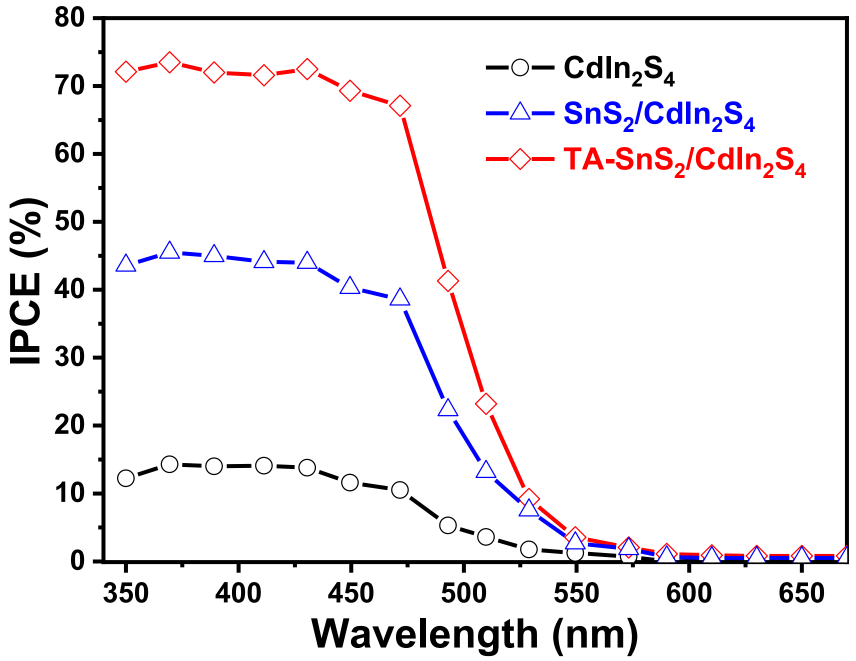

Figure S6. IPCE plots of $CdIn_2S_4$, $SnS_2/CdIn_2S_4$ and TA-$SnS_2/CdIn_2S_4$ photoanodes recorded at 1.23 V vs. RHE.

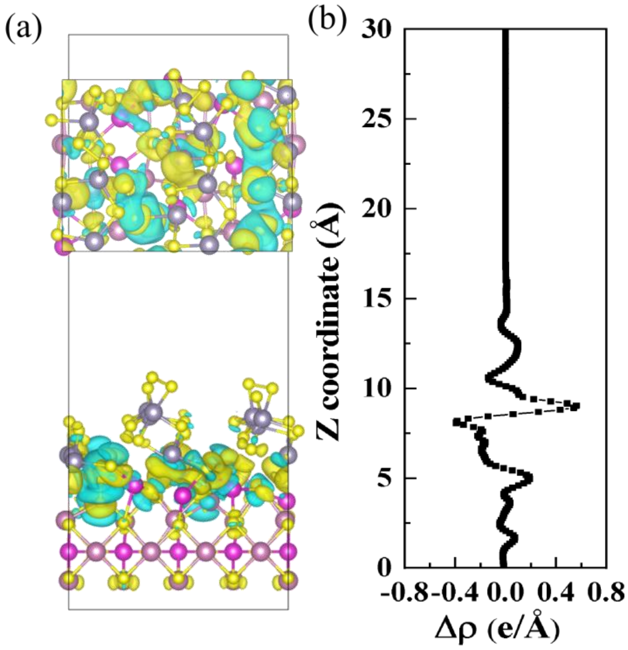

Figure S7. DFT calculations of the $SnS_2/CdIn_2S_4$ junction: (a) three-dimensional charge density difference (yellow, electron accumulation; cyan, electron depletion); (b) plane-averaged charge density difference along the z direction.

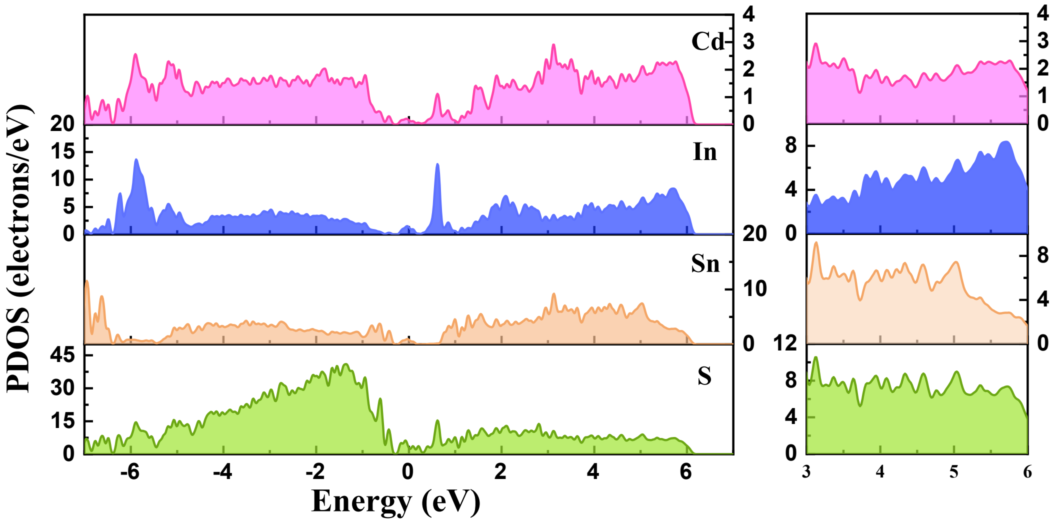

Figure S8. Projected density of states (PDOS) of the $SnS_2/CdIn_2S_4$ heterojunction.

Table S1. Comparison of the photocurrent densities (at 1.23 V vs. RHE) and ABPE values of the TA-SnS_2/$CdIn_2S_4$ photoanode with reported $CdIn_2S_4$-based photoanodes.

Photoanodes	Photocurrent (mA cm^{-2} at 1.23 V vs RHE)	ABPE (%)	refs
$CdIn_2S_4$/Cu_2O-20	7.51	2.72%	29
0.1Cu_2S/$CdIn_2S_4$	0.3	0.05%	30
$CdIn_2S_4$/InO_x/Ru	4.56	2.11%	31
TiO_2/$CdIn_2S_4$	0.68	0.16%	32
TiO_2/$CdIn_2S_4$-annealed	6.21	0.82%	32
$CdIn_{2.2}S_y$	Not reported	1.72%	33
V_s-$CdIn_2S_4$	5.73	2.49%	34
**TA-SnS_2/$CdIn_2S_4$**	**5.93**	**2.81%**	**This work**

Table S2. Illustrative biexponential fitting parameters for TRPL decay profiles.

**Sample**	**τ_1 (ns)**	**A_1 (%)**	**τ_2 (ns)**	**A_2 (%)**	**τ_{avg} (ns)**	**R^2**
$CdIn_2S_4$	0.668±0.024	86.3	1.150±0.038	13.7	0.771±0.019	0.975
$SnS_2/CdIn_2S_4$	0.706±0.033	87.3	1.280±0.041	12.7	0.825±0.025	0.964
TA-$SnS_2/CdIn_2S_4$	0.783±0.031	87.9	1.450±0.033	12.1	0.918±0.022	0.967

References

29. Wang, Q.; Hu, C.; Zhang, L.; Lei, Y.; Hu, Z.; Wang, Z.; Qiao, Y.; Lv, B. A highly efficient 3D/0D CdIn2S4/Cu2O photoanode with a p-n type heterojunction for boosted photoelectrochemical water splitting under visible light irradiation. J. Mater. Chem. C 2025, 13, 2790–2803.
30. Zhou, Z.L.; Liu, D.H.; Yang, B.; Wu, J.G.; Li, R. Utilizing the synergistic effect between p-n junction construction and field redistribution to achieve a high photocurrent density $Cu_2S/CdIn_2S_4$-based photoanode material for next-generation green clean energy. New J. Chem. 2025, 49, 18270–18281.
31. Chen, Z.Z.; Wang, L.; Jiao, X.L.; Xia, Y.G.; Chen, D.R. Thermally triggered surface reconstruction enables self-protective layer for enhanced charge transfer in $CdIn_2S_4$ photoelectrode. Chem. Eng. J. 2025, 167707.
32. Zhu, J.Y.; Cheng, Y.F.; Zhang, W.W.; Zhao, J.F.; Sun, Q.; Hu, X.Y.; Miao, H. Interfacial charge and surface defect regulation for high-efficiency $CdIn_2S_4$-based photoanodes. Appl. Surf. Sci. 2022, 601, 154188.
33. Sharma, M.D.; Basu, M. $CdIn_{2.2}S_y$ Nanosheet-Based Photoanodes for Photoelectrochemical Water Splitting. ACS Appl. Nano Mater. 2022, 5, 7811–7821.
34. Wang, H.M.; Xia, Y.G.; Li, H.P.; Wang, X.; Yu, Y.; Jiao, X.L.; Chen, D.R. Highly active deficient ternary sulfide photoanode for photoelectrochemical water splitting. Nat. Commun. 2020, 11, 3078.
